# Supplementary material for: PS1 Affects the Pathology of Alzheimer’s Disease by Regulating BACE1 Distribution in the ER and BACE1 Maturation in the Golgi Apparatus
Source: Int J Mol Sci. 2022 Dec 18;23(24):16151. doi: 10.3390/ijms232416151 (PMC9782474; doi:10.3390/ijms232416151)
Supplement: Supplementary file 1 [file ijms-23-16151-s001.zip › ijms-2059792-supplementary.pdf]

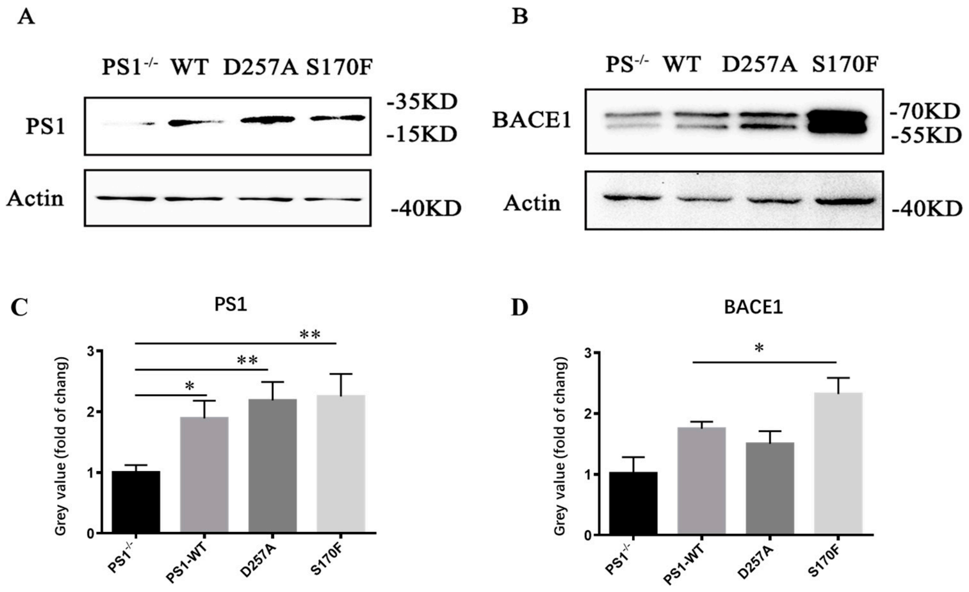

Figure S1. Western blot of lysates for 48 h transfected with pPS1-WT/pPS1-D257A/pPS1-S170F or without transfection in MEF PS1$^{-/-}$ cells. (A) The protein expression levels of PS1; (B) The protein expression levels of BACE1; Actin was used as reference; One-way ANOVA and Dunnett's post hoc test: $^{**}P < 0.01$,$^{*}P < 0.05$, (n = 3).

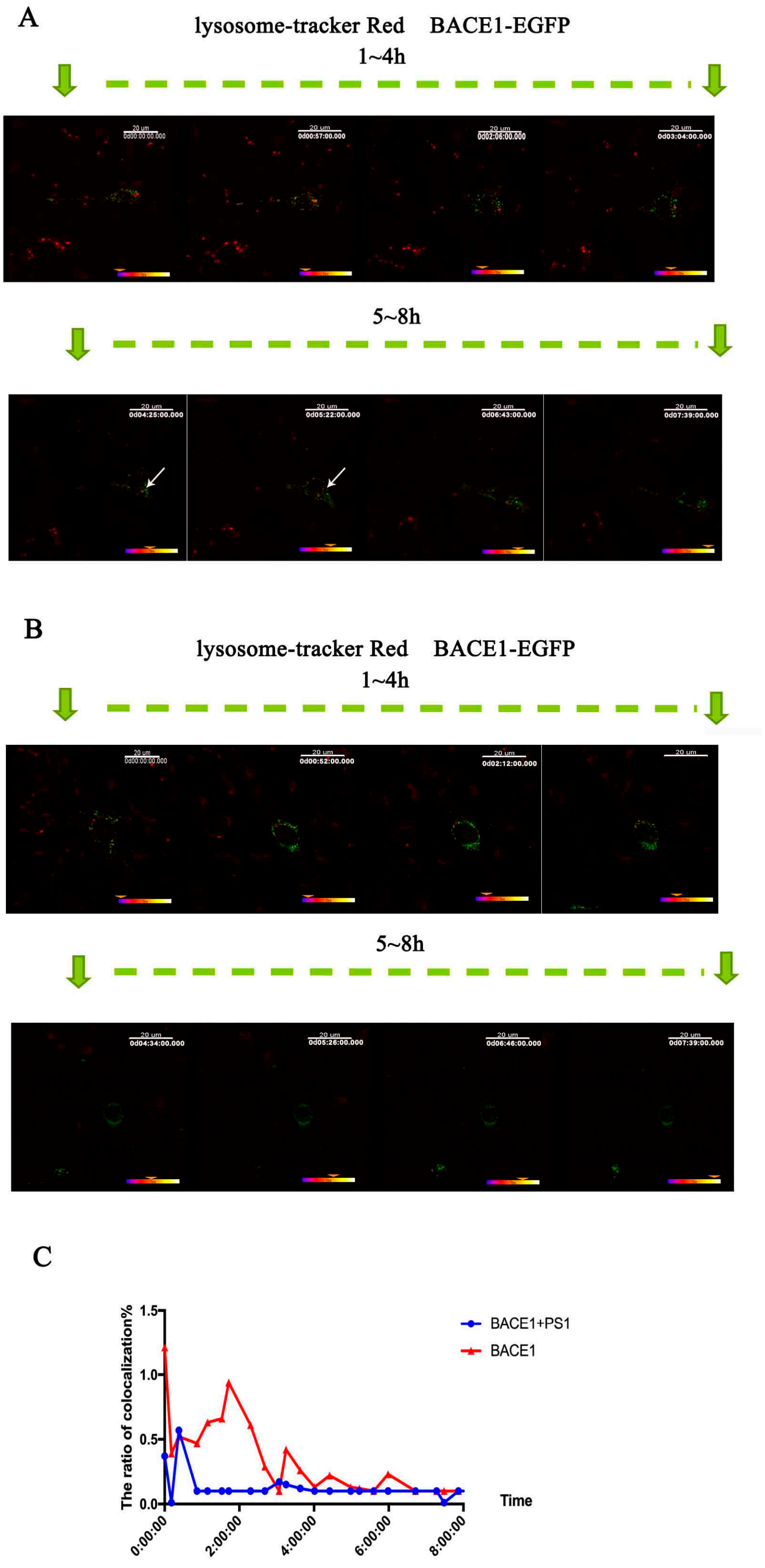

Figure S2. Time-lapse imaging of BACE1 and the lysosome tracker with or without PS1. (A)The distribution of BACE1-EGFP without PS1. (B) The distribution of BACE1-EGFP with PS1. (C) Quantitative analysis of the colocalization of BACE1-EGFP and the lysosome tracker with and without PS1.
